# Differential modulation of NMDA and AMPA receptors by cellular prion protein and copper ions

**DOI:** 10.1186/s13041-018-0406-3

**Published:** 2018-10-25

**Authors:** Sun Huang, Lina Chen, Chris Bladen, Peter K. Stys, Gerald W. Zamponi

**Affiliations:** 1 Department of Physiology and Pharmacology, University of Calgary, Calgary, AB Canada; 2 Department of Clinical Neurosciences, University of Calgary, Calgary, AB Canada; 3 Hotchkiss Brain Institute, University of Calgary, Calgary, AB Canada; 4 Alberta Children’s Hospital Research Institute, University of Calgary, Calgary, AB Canada

**Keywords:** NMDA receptor, AMPA receptor, Cellular prion protein, Knock-in mice, Whole-cell patch clamp, Hippocampal neurons, CNS disorders, Copper

## Abstract

N-Methyl-D-aspartate receptors (NMDARs) and α-amino-3-hydroxy-5-methyl-4-isoxazolepropionic acid receptors (AMPARs) are two major types of ionotropic glutamate receptors involved in synaptic transmission. However, excessive activity of these receptors can be cytotoxic and thus their function must be precisely controlled. We have previously reported that NMDA receptor activity is dysregulated following genetic knockout of cellular prion protein (PrP^C^), and that PrP^C^ regulation of NMDA receptors is copper-dependent. Here, we employed electrophysiological methods to study NMDAR and AMPAR currents of cultured hippocampal neurons from PrP^C^ overexpresser mice. We show that NMDA receptor current amplitude and kinetics are differentially modulated by overexpression of human or mouse PrP^C^. By contrast, AMPA receptor activity was unaffected. Nonetheless, AMPA receptor activity was modulated by copper ions in a manner similar to what we previously reported for NMDA receptors. Taken together, our findings reveal that AMPA and NMDA receptors are differentially regulated by PrP^C^, but share common modulation by copper ions.

## Introduction

Glutamate is the principal excitatory neurotransmitter in the mammalian central nervous system (CNS) and interacts with both metabotropic and ionotropic receptors [1] to trigger and modulate postsynaptic responses. Both NMDA and AMPA receptors are critical mediators of synaptic plasticity [2–7], whereas dysregulation of these receptors contributes to neurodegeneration in a wide range of disorders, including stroke and Alzheimer’s disease [8–14]. We have previously shown that cellular prion protein (PrP^C^) physically interacts with, and regulates NMDA receptor function [15, 16]. In hippocampal neurons, knockout of PrP^C^ leads to augmented NMDA receptor activity and a slowing of deactivation kinetics [17], potentially underpinning neurotoxicity and neurodegeneration. PrP^C^ is widely expressed across the nervous system and features four to five octapeptide repeats (depending on the species) in the unstructured N-terminal [18] which contain histidine residues that form multiple copper binding sites with varying copper affinities [19]. Chelation of copper ions with bathocuproine disulfonate (BCS) [20] results in increased NMDA receptor peak current amplitude, and a slowing of desensitization kinetics, leading to a large steady state NMDA current that may contribute to cytotoxicity. Here, we build on our previous findings to examine the effects of overexpressing PrP^C^ not only on NMDA receptors, but also on AMPA receptor activity. We show that overexpression of mouse and human PrP^C^ in hippocampal pyramidal cells resulted in altered NMDA receptor current amplitude and desensitization kinetics. AMPA receptor activity, on the other hand, does not appear to be affected by PrP^C^. AMPA receptors, however, are sensitive to copper chelation such that removal of copper ions resulted in increased steady state currents. Collectively, our data indicate distinct regulation of NMDA and AMPA receptors by PrP^C^, whereas both receptor types are similarly regulated by copper ions.

## Materials and methods

### Neuronal primary culture

Wild-type C57 mice were purchased from Charles River and maintained in compliance with the University of Calgary Animal Care and Use policies. Knock-in mice Tg650 (with human cellular prion protein) were provided by the French National Institute for Agricultural Research and Tga20 mice (with murine cellular prion protein) were provided by Dr. Frank Jirik and bred in house. Both mouse lines are originally from the European Mouse Mutant Archive. P0-P1 pups were used to prepare the hippocampal neurons for primary culture. All animal experiments were conducted with the approval of the animal care committee of the University of Calgary.

### Electrophysiology

Whole-cell voltage-clamp recordings were performed at room temperature on hippocampal pyramidal neurons after 10–15 days in culture, using an Axopatch 200B amplifier (Axon Instruments). The holding potential was − 60 mV throughout. The external solution contained 140 mM NaCl, 5 mM KCl, 1 mM CaCl_2_, 25 mM Hepes, and 33 mM D-Glucose, pH was adjusted to 7.4 with NaOH. To obtain NMDA currents, the external solution was supplemented with 15 μM 2,3-dihydroxy-6-nitro-7-sulfamoyl-benzo[f]quinoxaline (NBQX, from Tocris Bioscience), 100 μM picrotoxin (PTX), 1 μM tetrodotoxin (TTX, from Tocris Bioscience), 500 nM CuSO_4_ (to standardize [Cu^2+^] in external solution), and different concentrations of glycine as indicated. To obtain AMPA currents, the external solution was supplemented with 100 μM PTX, 1 μM TTX and 500 nM CuSO_4_. The internal pipette solution was composed of 140 mM CsCl, 11 mM EGTA, 1 mM CaCl_2_, 2 mM MgCl_2_, and 10 mM Hepes, pH was adjusted to 7.3 with CsOH. The internal solution was supplemented with 4 mM K_2_ATP and 0.6 mM GTP, added immediately before use. Superfusion was performed by a rapid microperfusion system (EVH-9, from Biologic Science Instruments) to achieve fast switching of solutions. The perfusion tip was positioned a few hundred micrometers from the cell and kept as constant as possible during the experiments. The solution exchange was computer controlled by a Digidata 1322A interface (Molecular Devices). NMDA receptor-mediated currents were evoked by application of 500 μM NMDA (Tocris Bioscience) and AMPA receptor-mediated currents were evoked by application of 100 μM AMPA (Tocris Bioscience). In a typical 30-s interval experiment, first external solution without agonist was applied to create a stable baseline, then the neuron was perfused with external solution with ligand for 7 s to evoke the currents, after reaching a stable state the channel was switched back to solution without ligand. The steady-state current was determined as the non-desensitizing current amplitude at the end of a 7-s application.

### Data analysis and statistics

Data were analyzed with either One-Way ANOVA with a Bonferroni post hoc test or via paired t-tests as appropriate. All error bars are S.E.M., and asterisks and number symbols denote statistical significance at the 0.05, 0,01, or 0.001 levels, respectively for one, two or three symbols.

## Results

We employed whole-cell voltage-clamp recordings to study the hippocampal neurons in C57 wild type, as well as Tg650 and Tga20 PrP^C^ knock-in mice over-expressing human and murine cellular prion protein, respectively.

### Human and mouse PrP^C^ differentially affects NMDA current activity

**Fig. 1:**
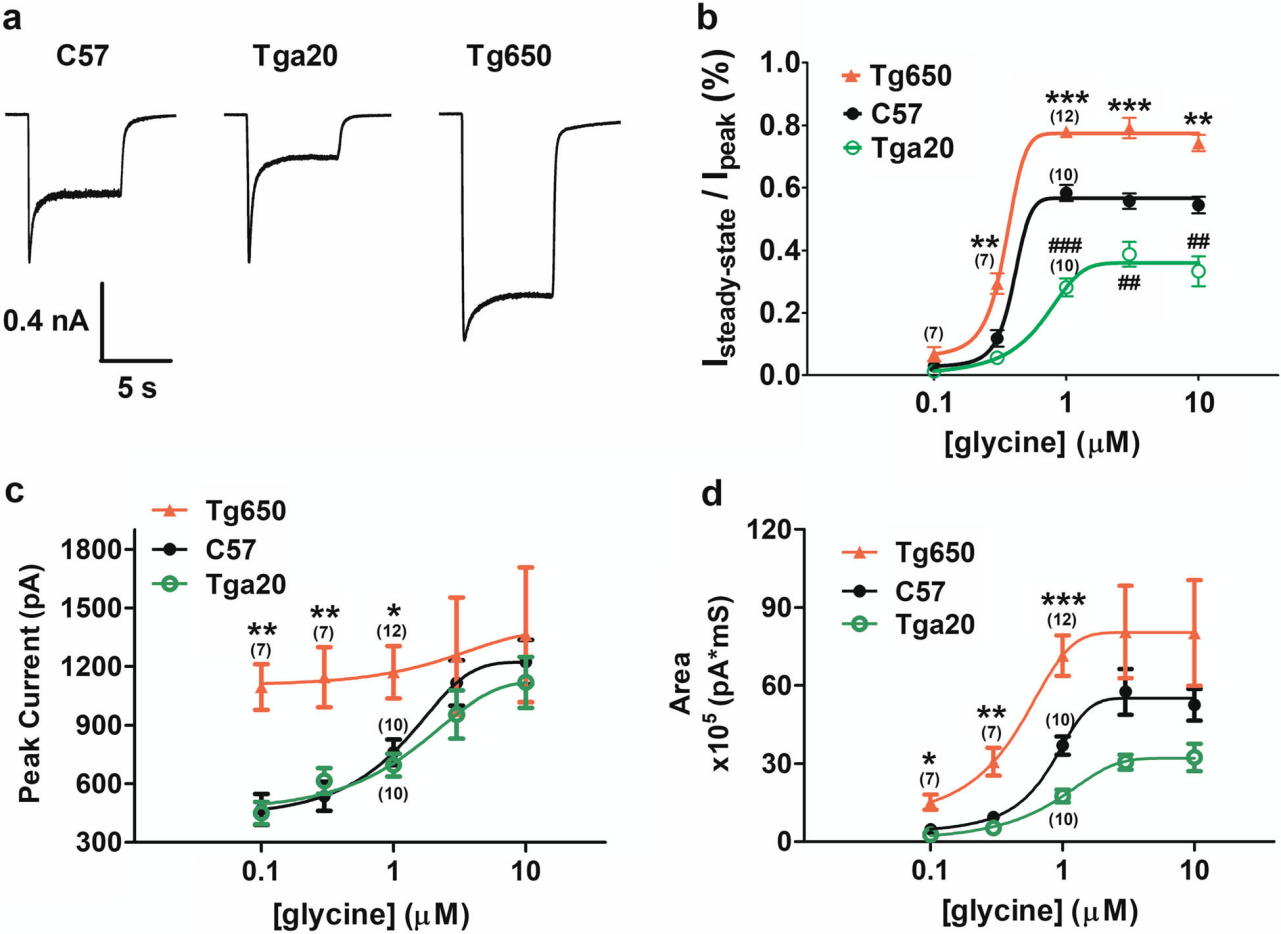
Modulation of NMDA receptors by cellular prion protein. **a**. NMDAR-mediated currents from hippocampal neuron cultures of Tga20 and Tg650 knock-in mice, versus wild-tpye C57. Neurons were held at − 60 mV throughout and currents were evoked by applciation of 500 μM NMDA, 1 μM glycine. **b**. Glycine dose response curve of the percentage of steady-state current (normalized to peak) in wild-type C57, Tga20 and Tg650 mice. **c**. Glycine dose response curve of the peak current in wild-type C57, Tga20 and Tg650 mice. **d**. Glycine dose response curve of the current area integration in wild-type C57, Tga20 and Tg650 mice. Unless stated otherwise, *n* = 5. Asterisks denote statistical significance for C57 vs Tg650 data, and number symbols indicate statistical significance between C57 and Tga20 (one way ANOVA)

NMDA receptors require the co-agonist glycine for channel activation, in addition to the primary agonist glutamate or NMDA, with increasing glycine concentrations resulting in both an increase in whole cell NMDA current amplitude, and a slowing of desensitization kinetics. Figure 1a depicts typical agonist-evoked NMDA currents in the presence of 1 μM glycine. As evident from the raw current data, the overexpression of mouse PrP^C^ leads to increased desensitization compared to those observed in C57 mouse neurons, as reflected in a decrease in steady state current. Overexpression of human PrP^C^ produced the opposite effect leading to less desensitization of NMDA currents in the prolonged presence of agonist. Figure 1b examines steady state (i.e. non desensitizing current) of the receptors for the three mouse lines in response to a range of glycine concentrations. Here, the steady state NMDA current was normalized to the peak current amplitude, and therefore values close to 1 correspond to completely non-desensitizing currents, whereas zero reflects complete desensitization. As evident from the figure, NMDA receptors in Tg650 neurons showed increased glycine sensitivity and much less complete desensitization compared to Tga20 or wild type mice. The NMDA receptor peak current amplitude of Tga20 and wild type mice was similar across the entire range of glycine concentrations, whereas that of Tg650 was augmented at lower glycine concentrations, suggesting an overall enhancement of glycine sensitivity of NMDA receptors co-expressed with an excess of human PrP^C^ (Fig. 1c). The area under the current traces, representing the total charge admitted into the cell during the period of agonist application, is a combined measure of peak and steady-state currents. As shown in Fig. 1d, the pattern across the three strains at various glycine concentrations paralleled that of the steady-state currents in Fig. 1b. NMDA receptors from Tg650 mouse neurons had a higher sensitivity to glycine and admitted more charge during the period of agonist application, compared to WT neurons. The opposite was true for Tga20 neurons.

### AMPARs are weakly regulated by PrP^C^

**Fig. 2:**
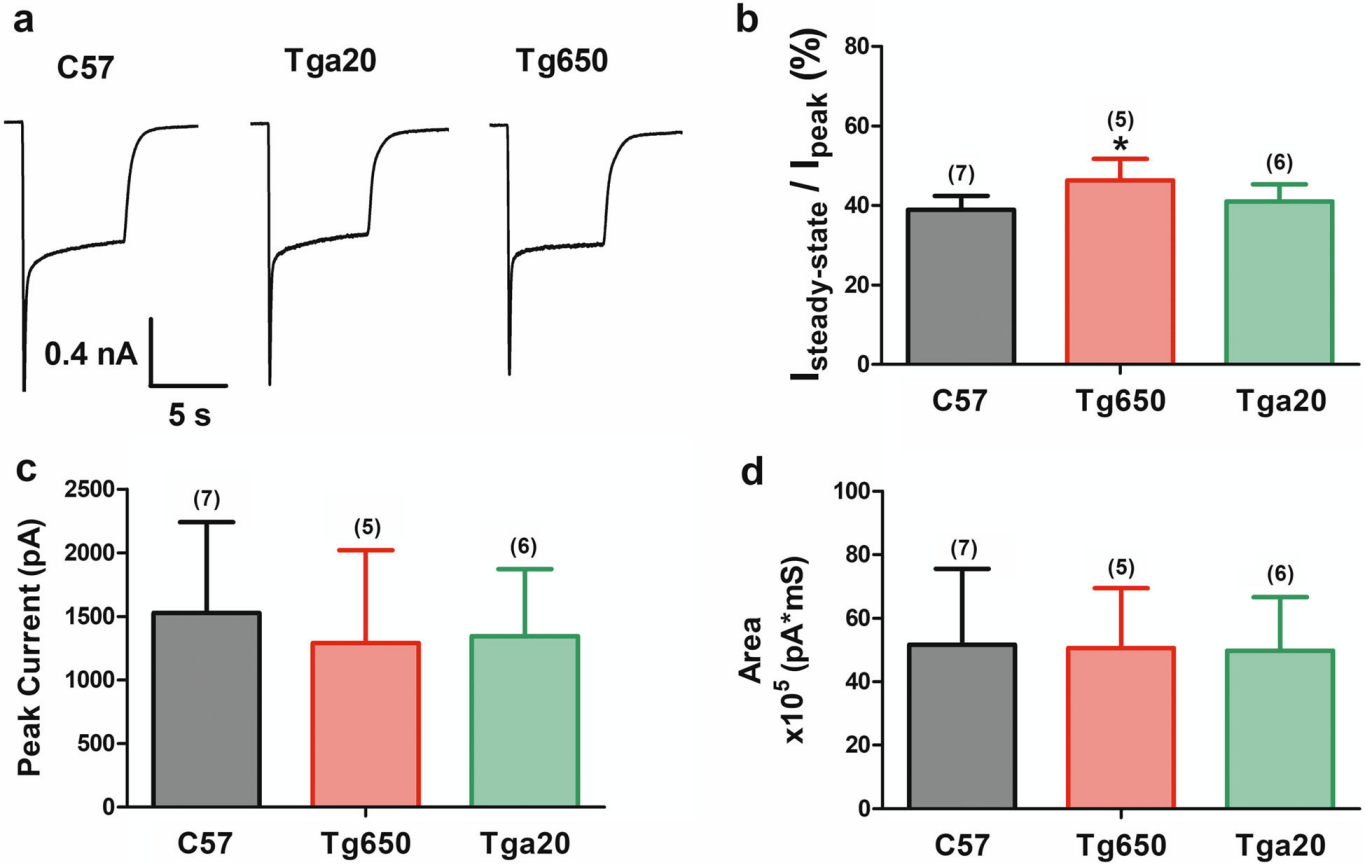
Modulation of AMPA receptors by cellular prion protein. **a**. Representative traces of AMPAR-mediated current from hippocampal neurons of C57, Tga20, and Tg650 mice. Neurons were held at − 60 mV throughout and currents evoked by application of 100 μM AMPA. **b**. Steady-state AMPA current (normalized to peak) in neurons from C57, Tga20 and Tg650 mice. **c**. Comparison of AMPAR peak currents. **d**. Comparison of AMPAR current area integration. Asterisks denote statistical significance for C57 vs Tg650 data (one way ANOVA)

We then performed analogous experiments on AMPA receptors expressed in Tga20, Tg650 and C57 mouse hippocampal neurons. Unlike NMDA receptors, no co-application of glycine was needed. As evident from Fig. 2, overexpression of either mouse or human PrP^C^ did not strongly affect desensitization kinetics (Fig. 2a, b), nor did it alter peak current amplitude or overall cation influx (Fig. 2b-d). Only AMPA receptors from Tg650 neurons exhibited a modest but significantly increased steady-state current. These data indicate that AMPA receptors are not as strongly regulated by overexpression of different PrP^C^ species compared to what is seen with NMDA currents.

### AMPA receptor activity is copper dependent

**Fig. 3:**
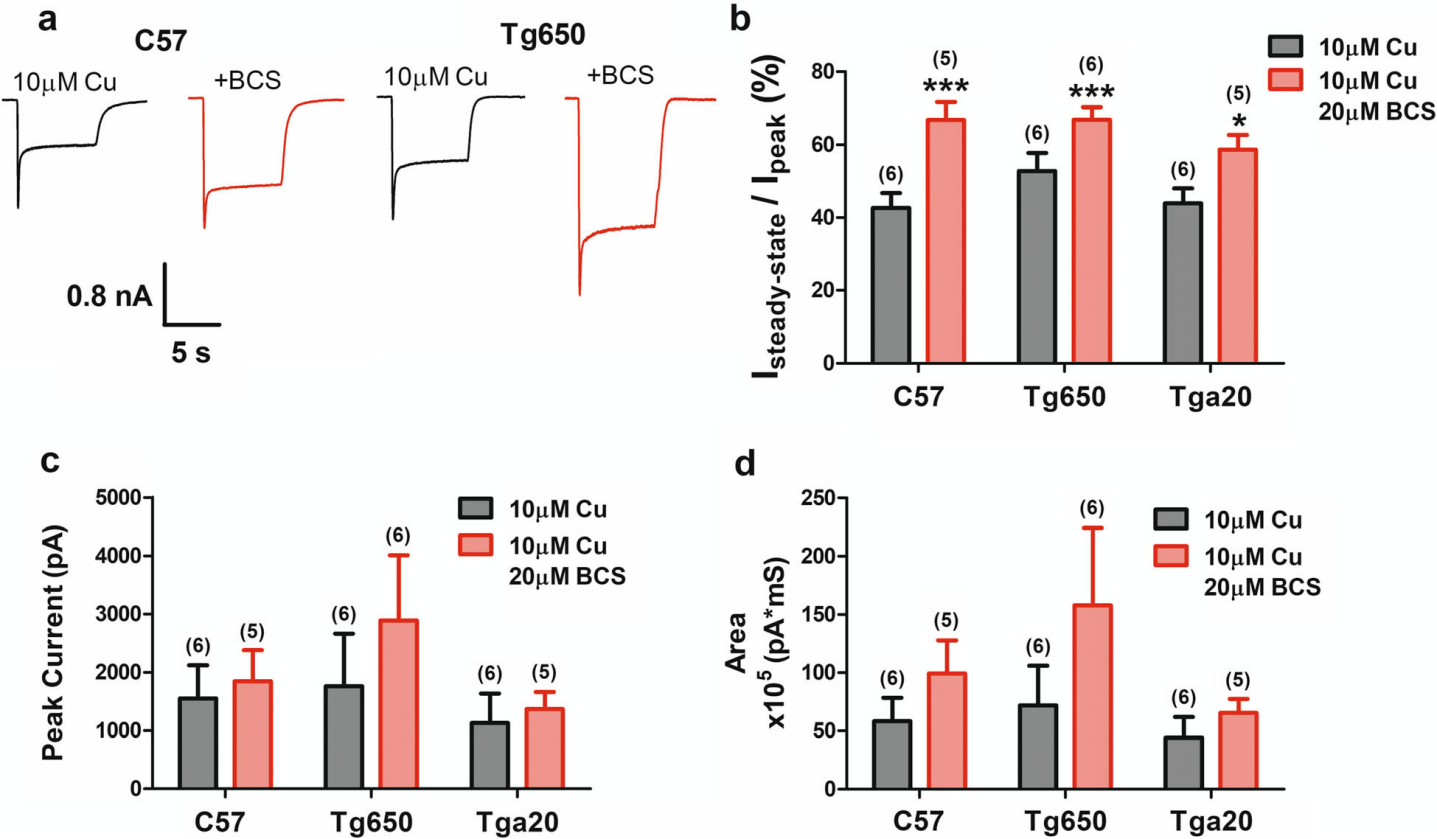
Modulation of AMPA receptors by copper ions. **a**. sample traces of AMPA receptor currents in C57 and Tg650 neurons in either 10 μM copper, or 10 μM copper + 20 μM BCS (nominally zero free copper). **b**. Steady state AMPA current (normalized to peak) obtained from C57, Tga20 and Tg650 mouse hippocampal neurons in the presence of 10 μM copper, or 10 μM copper plus 20 μM BCS. **c**. Comparison of peak currents before and after BCS application as in panel **a**. **d**. Integrated current area under the conditions of panels **a** and **b**. Asterisks denote statistical significance (paired t-test)

Previous work revealed both PrP^C^-dependent and independent regulation of NMDA receptors by copper ions [15, 16]. To determine if copper ions are also able to regulate AMPA receptor activity, we performed experiments where extracellular copper was clamped at 10 μM, and then chelated by an excess of 20 μM BCS which is highly selective for this metal and exhibits an affinity in the attomolar range (thus allowing us to compare the effects of 10 μM copper to a nominally copper free condition). Figure 3a and b shows that copper chelation results in an increase in steady-state AMPA current for both Tg650 and wild type neurons, to similar levels (as a ratio in comparison with copper-replete traces) across the three mouse lines. These data also unmask a small difference in AMPA current desensitization in 10 μM copper between C57 and Tg650 neurons, with the latter exhibiting a modest but significantly greater steady-state current (Fig. 3b). There was only a small effect of copper chelation on peak current amplitude and total cation entry in the two experimental conditions that appeared to be somewhat larger in Tg650 neurons (Fig. 3c and d). Overall, these data indicate that copper has the ability to regulate AMPA receptors, in a manner that is qualitatively similar to what we had reported previously for NMDA receptors.

## Discussion

Here we report that two types of ionotropic glutamate receptors are differentially modulated by overexpression of either mouse or human PrP^C^. Additionally, the kinetics of both receptors are regulated by copper ions. This work builds on two previous studies from our groups. The first study reported the effects of depleting PrP^C^ on whole cell NMDA currents in mouse hippocampal neurons. These early findings revealed that the absence of PrP^C^ slowed NMDA receptor deactivation and drastically augmented the magnitude of NMDA receptor mediated synaptic events, whereas AMPA receptor-mediated events showed a small enhancement of current amplitude, but no change in rise or decay times [17]. In a second study, we examined the effects of copper chelation on NMDA receptor currents in rat and in C57 mouse hippocampal neurons. These experiments revealed an augmentation of peak current amplitude and steady state NMDA current upon copper chelation. They also showed that NMDA currents in PrP^C^ null mouse neurons exhibited a leftward shift in the glycine dose-response of steady state current, so that lower levels of ambient glycine were required for receptor activation. Here, (see Fig. 1b) overexpression of mouse PrP^C^ (roughly six-fold in Tga20 mice compared to C57) mediated the opposite effect where a rightward shift in the glycine response curve and a reduced plateau of steady state current reflected a greater desensitization of NMDA currents. These findings are consistent with an overall protective role of PrP^C^ that could function to reduce overall cation flux through these receptors and subsequent excitotoxicity. Remarkably, overexpression of human PrP^C^ (roughly six fold in Tg650 mice over PrP^C^ levels in C57) [21] had an opposite effect compared to mouse PrP^C^, resulting in increased sensitivity to glycine of NMDA receptor-mediated currents. In some ways, our data with Tg650 neurons are reminiscent of what we had observed previously with PrP^C^ null mice, suggesting that either human PrP^C^ cannot effectively interact with mouse NMDA receptors, or that human PrP^C^ does not bear the ability to functionally regulate receptor desensitization. Human and mouse PrP^C^ are approximately 90% identical at the amino acid levels, with minor differences occurring throughout the entire length of the protein (including small deletions and insertions). It is known that even single amino acid changes can affect the folding and misfolding of PrP^C^ and it is possible that the NMDA receptor is differentially sensitive to the conformation adopted by human and mouse PrP^C^. This could explain the late neurodegeneration that occurs spontaneously in aged Tg650 mice [22], resulting from a subtle but chronic excitotoxicity and excessive calcium loads experienced by these neurons over time. Moreover, these observations support the notion that misfolded PrP as occurs in a variety of prionopathies, could become less competent in its ability to regulate NMDA receptor kinetics, likewise leading to excitotoxicity and neuronal death.

In contrast to NMDA receptors, AMPA receptors do not appear to be strongly regulated by overexpression of mouse or human PrP^C^, and these data are consistent with our earlier synaptic work in PrP^C^ null mouse neurons that showed only a small effect on miniature synaptic events in hippocampal cultures [17]. This suggests that PrP^C^ may either not be able to interact with AMPA receptors at all, or alternatively, that the interactions may simply not produce potent functional effects. The observation that there was a small but statistically significant effect on steady state current in Tg650 neurons may support the latter possibility. There was, however, a potent effect of copper chelation on steady-state current amplitude. It is unclear whether this effect is mediated by indirect interactions via the copper binding sites on PrP^C^, other interacting copper-binding proteins, or by copper ions interacting with the pore of the receptor directly. Indeed, we note that copper ions are able to speed desensitization and inhibit peak current amplitude of NMDA currents in PrP^C^ null mice, consistent with open channel block, and a similar mechanism may be at play here with AMPA receptors. Acute effects of copper on AMPA-mediated synaptic events have been reported in the literature, and are consistent with our present findings [23].

Altogether, our findings reveal differences in the regulation of AMPA and NMDA receptor by cellular prion protein. In the context of prion disease, synaptic deficits due to misfolding of PrP^C^ are thus more likely to arise from dysregulation of NMDA receptors than AMPA receptors. Our data also reinforce the central role of copper ions as potent regulators of two major excitatory receptors in the CNS.

## Abbreviations

AMPA: α-amino-3-hydroxy-5-methyl-4-isoxazolepropionic acid
BCS: Bathocuproine disulfonate
NMDA: N-Methyl-D Aspartate
PrP^C^: Cellular prion protein
PTX: Picrotoxin
TTX: Tetrodotoxin
